# Depression in Sjögren’s syndrome mediates the relationship between pain, fatigue, sleepiness, and overall quality of life

**DOI:** 10.2478/rir-2023-0012

**Published:** 2023-07-22

**Authors:** Tiago Costa, Stephen P Rushton, Stuart Watson, Wan-Fai Ng, On behalf of the UK primary Sjögren's Syndrome Registry

**Affiliations:** Translational and Clinical Research Institute, Faculty of Medical Sciences, The Medical School, Newcastle University, Framlington Place, Newcastle upon Tyne, NE2 4HH, UK; Northern Centre for Mood Disorders, Wolfson Research Centre, Campus for Ageing and Vitality, Newcastle University, Newcastle upon Tyne, NE4 5PL, UK; Cumbria, Northumberland, Tyne and Wear NHS Foundation Trust, St. Nicholas Hospital, Jubilee Road, Gosforth, Newcastle upon Tyne, NE3 3XT, UK; School of Natural and Environmental Science, Agriculture Building, Newcastle University, King’s Road, Newcastle upon Tyne, NE1 7RU, UK; National Institute for Health and Care Research (NIHR) Newcastle Biomedical Research Centre, Biomedical Research Building, Campus for Ageing and Vitality, Newcastle upon Tyne, NE4 5PL, UK; NIHR Newcastle Clinical Research Facility, Level 6, Leazes Wing, Royal Victoria Infirmary, Newcastle upon Tyne Hospitals NHS Foundation Trust, Queen Victoria Road, Newcastle upon Tyne, NE1 4LP, UK

**Keywords:** Sjögren’s syndrome, depression, fatigue, quality of life, autonomic function

## Abstract

**Objectives:**

Sjögren’s syndrome (SS) includes many extra-glandular symptoms such as fatigue, pain, sleepiness and depression, which impact on quality of life (QoL). These symptoms also influence each other and could be linked by autonomic nervous system (ANS) dysregulation. Our aim was to model the role of putative predictive variables, including depression in the relationships between ANS function, fatigue, and QoL in SS.

**Methods:**

Cross-sectional analysis of self-reported data from the multicentre UK primary SS registry. The Composite Autonomic Symptom Scale (COMPASS) was used to assess autonomic function, the Hospital Anxiety and Depression Scale (HADS) to assess anxiety and depression and the EuroQol-5 Dimension (EQ-5D) to assess QoL. Validated scales were used for other clinical variables. Using multiple regression analysis and structural equation modelling (SEM), we investigated how the QoL of people with SS is impacted by the direct and indirect effects of fatigue, sleepiness, depression, symptom burden and ANS function, and their interactions.

**Results:**

Data was obtained for 1046 people with SS, 56% COMPASS completers. Symptoms of ANS dysregulation were common. Participants with ANS dysregulation had more severe depression, anxiety, dryness, fatigue, pain, sleepiness and QoL (*P* < 0.01 for all). Depression, anxiety, dryness, and pain were independent predictors of ANS function in the multiple regression model (*P* < 0.05 for all). ANS function could not be included in the SEM. The SEM model had good fit to the data (comparative fit index = 0.998) and showed that, in people with SS, depression mediates the effects of pain, fatigue and sleepiness on QoL.

**Conclusion:**

Our results show that diagnosing and treating depression in people with SS could have direct positive impact on QoL, and significantly ameliorate the impact of fatigue and pain.

## Introduction

Sjögren’s syndrome (SS) is a systemic autoimmune disease characterized by dryness due to dysfunction of the lachrymal and salivary glands. Ocular and oral dryness characterize the disease and can be understood as a proxy for glandular disease activity or severity. Extra-glandular involvement is also common, including fatigue,^[1,2]^ musculoskeletal pain,^[3]^ neurologic symptoms,^[3]^ and depression.^[4]^ People with SS have significant functional disability when compared to age-matched healthy controls and this is associated with reduced quality of life (QoL).^[5]^

Fatigue is a arguably the most burdensome symptom for people with SS,^[6]^ having a significant impact on work productivity^[7]^ and QoL.^[1]^ The pathophysiology of fatigue in SS is not fully understood. Fatigue^[8]^ and work disability^[7]^ correlate poorly with serological disease activity. On the other hand, fatigue correlates more strongly with symptoms such as dryness,^[9]^ daytime sleepiness,^[9]^ pain,^[8,9]^ cognitive dysfunction^[10]^ comorbid depression.^[1,11]^

Autonomic nervous system (ANS) dysregulation is estimated to occur in 55% of people with SS, significantly more than age- and sex-matched controls.^[2]^ ANS dysregulation correlates with fatigue,^[2,12]^ disease activity,^[2]^ symptom burden,^[13]^ pain,^[14]^ co-morbid depression^[12]^ and QoL.^[13]^ It has been proposed that ANS dysregulation could relate to the underlying SS pathology.^[15]^ Like SS, depression is known to be associated with ANS dysregulation^[16]^ and is significantly more prevalent in people with SS than in healthy controls.^[4]^ Therefore, ANS dysregulation could link the different clinical features of SS, including comorbid depression.

Understanding how all these factors impact on the QoL of people with SS is complicated by the fact that many of the symptoms may be linked and influence each other. Whilst it is possible to relate QoL to the levels of individual symptoms, this does not help us understand their overall impacts. For example, fatigue can exacerbate ocular symptoms^[17]^ and there is evidence that nocturnal humidification devices improve sleep in people with SS,^[6]^ suggesting that treating dryness can have the indirect effect of reducing daytime sleepiness and fatigue.

Structural equation modelling (SEM) provides a framework to investigate how different potential drivers impact in their effects on overall QoL. SEM is an extension of pathway analysis that seeks to quantify the direct effects that an individual driver may have on an outcome, but also the indirect effects it may have through its impact on other drivers. The approach is based on using data to challenge a priori conceptual model(s) of how the drivers impact on each other and the outcome. The relationships between drivers are characterized by a series of equations that link the outcomes to one or more predictors that are defined a priori. The goodness of fit of the conceptual pathway model to the data is assessed through analysis of the variance and covariance structure of the putative relationships in the network of pathways.

We hypothesise that ANS dysregulation is significantly and positively correlated with co-morbid depression in people with SS, and that depression has a significant negative impact on levels of fatigue and QoL. Therefore, we hypothesise that people with SS and comorbid depression have worse indices of fatigue and QoL than their peers with similar indices of disease activity. In this study our objective is to model the role of depression on the relationship between ANS function, fatigue, and QoL in SS, including other predictive variables. We use SEM to investigate how the QoL of people with SS is impacted by the direct and indirect effects of fatigue, sleepiness, depression, symptom burden and ANS function, and their interactions.

## Methods

### Patients

All patient data were extracted from the UK Primary Sjögren’s syndrome Registry (UKPSSR, www.sjogrensregistry.org).^[18]^ The UKPSSR is a research biobank of people with SS, based on a multicentre, cross-sectional study, collecting standardized patient-reported measures and clinical-generated assessments of people with SS from across the UK. In this analysis we included assessments completed between August 13, 2009 and February 28, 2019. All participants in the UKPSSR provided informed consent and fulfilled the American European Consensus Group (AECG) classification criteria for SS.^[19]^ A primary diagnosis of fibromyalgia was an explicit exclusion criterion for the UKPSSR, to reduce potential bias on the assessment of pain and fatigue, as fibromyalgia is a recognised comorbidity in people with SS but is believed to have a different pathophysiology. Research ethics approval was granted by the North West Research ethics committee in the UK.

### Data and Instruments

The methods for clinical and laboratory data collection for the UKPSSR have been described.^[18]^ Briefly, all clinical and laboratory data were collected prospectively using a standardised pro forma at time of recruitment. Standardised patient-reported outcome measures (PROMs) were used for assessment of the different symptom domains in this analysis. The Composite Autonomic Symptom Scale (COMPASS)^[20]^ was used to assess the severity of ANS symptoms. The COMPASS consists of 73 questions divided into 10 system domains: orthostatic intolerance, vasomotor, secretomotor, gastroparesis, autonomic diarrhoea, constipation, bladder, pupil and focusing, sleep disorder and syncope. The optional male erectile dysfunction domain was not included, as SS predominantly affects females. Each domain is scored in relation to the presence, severity, distribution, frequency, and progression of symptoms. The domain scores are then weighted, and the total sum is an indication of overall autonomic symptom burden, which we used for this analysis. A higher score is indicative of increased severity in autonomic dysregulation. A COMPASS score of 32.5 has been identified as a cut-off value for ANS dysregulation in people with chronic fatigue syndrome.^[21]^ The EuroQol-5 Dimension (EQ-5D)^[22]^ was used to assess health-related QoL, including a visual analogue score of overall health state, from 0 to 100, with 100 being the best imaginable health state. The result from the EQ-5D visual analogue scale (EQ-5D-VAS) was used for this analysis, as the primary outcome. The Epworth Sleepiness Scale (ESS)^[23]^ includes 8 questions assessing sleepiness, on a 0-3 scale, which are then summed. A higher score is indicative of increased daytime sleepiness. The total score was used to assess sleepiness in this analysis. The European Alliance of Associations for Rheumatology (EULAR) Sjögren’s Syndrome Patient Reported Index (ESSPRI)^[24]^ was used to assess the overall symptom burden. The ESSPRI consists of a simple 0-10 numerical scale each for dryness, pain, and fatigue. The ESSPRI score is the average of the three sub-scores. Its construct validity has been demonstrated.^[25]^ A higher score is indicative of higher overall symptom burden. The ESSPRI pain scale was used to measure pain in this analysis. The oral and ocular dryness scores (0-10 numerical scale) were used to calculate the EULAR sicca score (EULAR-SS), a measure of overall severity of dryness experienced by people with SS patients and defined as ([2 x oral dryness + ocular dryness] /3). The Hospital Anxiety and Depression Scale (HADS)^[26]^ was used to assess anxiety and depression. It has 7 items for anxiety (HADS-A) and 7 items for depression (HADS-D), scored 0-3 and then summed. Higher scores are indicative of increased severity. The Orthostatic Grading Scale (OGS)^[27]^ consists of a 5-item questionnaire for frequency and severity of orthostatic symptoms, relationship to orthostatic stressors, impact on activities of daily living and standing time. Higher scores are indicative of increased severity. The Profile of Fatigue (PROF)^[28]^ was developed specifically for people with SS. It includes 6 questions assessing somatic and mental fatigue, on a 0-7 scale. An average is then taken for each domain. The score from the PROF somatic fatigue (PROF-SF) domain scale was used for this analysis, with higher scores indicative of increased severity. Completion of the COMPASS was opt-in; all other PROMs were mandatory, although only those in which there was no missing data were used for the SEM analysis.

### Regression Analysis

To identify independent predictors of COMPASS scores, multivariate stepwise linear regression was performed using the COMPASS score as the dependent variable. The PROMs of interest-EQ-5D-VAS, ESS, ESSPRI, EULAR-SS, HADS-D, HADS-A, OGS, and PROF-SF-were inputted as independent variables. The F-statistic was used for the stepping model, with entry at 0.05 and removal at 0.10. Cases with missing data were excluded pairwise. The model produces standardised coefficients (β) for each independent predictor variable, which allow us to compare how many standard deviations the COMPASS score changes for a standard deviation change in any given predictor variable. We used these methods to directly compare our results with a previous analysis of ANS symptoms on UKPSSR data.^[2]^

### Conceptual Model

**Figure 1:**
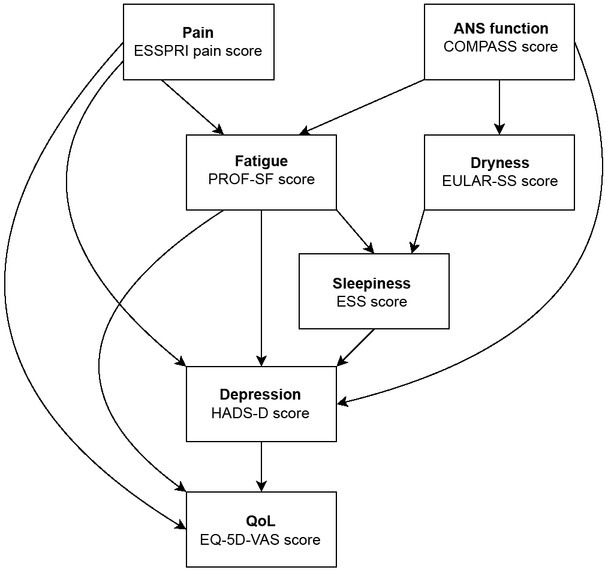
Conceptual model outlining hypothesised putative relationships between various health and symptoms of people with SS and their impacts on overall QoL. EQ-5D-VAS, EuroQol-5 dimension health-related quality of life scale, visual analogue scale; ESS, epworth sleepiness scale; ESSPRI, European alliance of associations for rheumatology (EULAR) Sjögren’s syndrome patient reported index; HADS-D, hospital anxiety and depression acale, depression sub-scale; PROF-SF, profile of fatigue and discomfort, somatic fatigue scale; QoL, quality of life.

The conceptual model of the symptom outcome interactions was derived from hypothesised direct and indirect effects as reported in prior research. We hypothesised that QoL was directly influenced by patient symptom burden (pain and dryness),^[29]^ the severity of depression,^[2]^ fatigue^[29]^ and ANS dysfunction.^[13]^ However, patient symptom burden is also likely to impact on the level of fatigue experienced by a person with SS^[8,9]^ and the severity of depression.^[9]^ Following on from this, fatigue is likely to impact on sleepiness.^[9]^ Sleepiness by the same token could also impact on depressive symptoms.^[9]^ This leads to a model describing the hypothesised pathways of how different symptoms of the disease contribute to the overall QoL of people with SS (Figure 1).

### Structural Equation Model (SEM)

We used standardised measures for each of the parameters (Figure 1) as inputs to challenge our conceptual model as a SEM. We fitted the model using Version 0.6-15 for lavaan library and version 4.3.0 for R.^[30]^ We first fitted a full model with all putative explanatory relationships in the pathway model. Models were assessed on the basis of two indices of fit, the Root Mean Square Error of Association (RMSEA) and the Comparative Fit Index (CFI). An RMSEA of less than 0.05 indicates that the data was a good fit to the hypothesised model of the relationships between symptoms and QoL outcomes. A CFI index of 0 indicates a complete lack of fit, whilst an index of 1 indicates a complete match between the conceptual model and the data used to challenge it. SEM also generates the equivalent of regression and standardised coefficients which allow estimation of the significance and magnitude of the effects of the individual hypothesised pathways. Non-significant pathways were removed from the model until only those variables having significant effects remained. This stepwise removal of non-significant variables led to a final ‘parsimonious’ model which can be used to quantify the relative importance of direct and indirect effects on the QoL outcome.

## Results

### Patient Characteristics

At the time of analysis, 1046 people with SS had been recruited to the UKPSSR: 90% were female, 91% Caucasian and the mean age was 58.1 years. Table 1 provides a summary of socio-demographic characteristics and descriptive statistics for scores of PROMs of interest: COMPASS, EQ-5D-VAS, ESS, ESSPRI, EULAR-SS, HADS-A, HADS-D, OGS, and PROF-SF.

**Table 1:** Clinical characteristics of sample

Gender, *n* (%)	
*n*	1011
Female	940 (90)
Age, years	
*n*	1034
Mean (SD)	58.1 (12.8)
Ethnicity, *n* (%)	
*n*	1046
Caucasian	947 (91)
Indian	22 (2)
Black	16 (1.5)
Other	61 (5.5)
BMI, kg/m^2^	
*n*	986
Mean (SD)	26.7 (5.9)
ANS function, COMPASS total weighted score	
*n*	627
Mean (SD)	34.7 (18.2)
Anxiety, HADS-A score	
*n*	969
Mean (SD)	7.9 (4.6)
Depression, HADS-D score	
*n*	969
Mean (SD)	5.8 (4.0)
Dryness, EULAR-SS score	
*n*	984
Mean (SD)	5.9 (2.5)
Fatigue, PROF-SF score	
*n*	981
Mean (SD)	3.8 (1.7)
Pain, ESSPRI pain scale	
*n*	986
Mean (SD)	5.4 (2.2)
Orthostatic intolerance, OGS score	
*n*	929
Mean (SD)	3.7 (3.5)
QoL, EQ-5D-VAS score	
*n*	975
Mean (SD)	60.4 (21.3)
Sleepiness, ESS score	
*n*	944
Mean (SD)	8.6 (4.9)

BMI, body mass index; COMPASS, composite autonomic symptom scale; EQ-5D-VAS, EuroQol-5 dimension health-related quality of life scale, visual analogue scale; ESS, epworth sleepiness scale; ESSPRI, European alliance of associations for rheumatology (EULAR) Sjögren’s syndrome patient reported index; EULAR-SS, European alliance of associations for rheumatology sicca score; HADS-A, hospital anxiety and depression scale, anxiety sub-scale; HADS-D, hospital anxiety and depression scale, depression sub-scale; kg, kilograms; m, meters; OGS, orthostatic grading scale; PROF-SF, profile of fatigue and discomfort, somatic fatigue scale; SD, Standard Deviation; QoL, quality of life.

### Autonomic Function

From the total sample of 1046 people with SS, 627 (60%) opted-in for the COMPASS assessment. On average, data for other variables was missing in 2% of COMPASS completers and 12% of non-completers (*n* for each variable on Table 2). Gender and age were similar for COMPASS completers and non-completers, but there was a higher proportion of Caucasians amongst COMPASS completers (the observed count of Caucasian COMPASS completers was 596 and the expected count was 569, *chi-squared P* < 0.001). When comparing COMPASS completers and non-completers, the completers scored significantly less severely (all *P* < 0.05 on *t*-test) on the PROF-SF (mean score 3.7 *vs*. 4.0), ESSPRI pain scale (mean score 5.3 *vs*. 5.7) and OGS scores (mean score 3.5 *vs*. 4.1), but effect sizes were small (Cohen’s d 0.16 to 0.21, confidence intervals on Table 2). Descriptive statistics for COMPASS completers and non-completers are shown in Table 2.

**Table 2:** Clinical characteristics for COMPASS non-completers and completers

	COMPASS completers *n* = 628	COMPASS non-completers *n* = 418	Effect of group	
			Comparator test	Effect size (95% CI)
Gender, *n* (%)				
*n*	628	383	*P* = 0.298^a^	0.03^b^
Female	588 (94)	352 (92)		
Age, years				
*n*	626 58.1	408 58.2	*F* = 0.511 *t* = 0.124	0.08 (-0.12, 0.13)^d^
Mean (SD)	(12.5)	(13.2)	*P* = 0.902^c^	
Ethnicity, *n* (%)				
*n*	628	418		
Caucasian	596 (95)	351 (84)	*P* < 0.001^a^	0.25^b^
Indian	12 (1.9)	10 (2.4)		
Black	5 (0.8)	11 (2.6)		
Other	15 (2.3)	46 (11)		
BMI, kg/m^2^				
*n*	622	364	*F* = 2.079 *t* = 0.269	
Mean (SD)	26.6 (5.6)	26.8 (6.3)	*P* = 0.788^c^	0.02 (-0.11, 0.15)^d^
ANS function, COMPASS total weighted score				
*n*	628	418	-	-
Mean (SD)	34.7 (18.2)	-		
Anxiety, HADS-A score				
*n*	612 7.8	356 7.9	*F* = 0.252 *t* = 0.353	0.02 (-0.11, 0.15)^d^
Mean (SD)	(4.5)	(4.6)	*P* = 0.724^c^	
Depression, HADS-D score				
*n*	615	354	*F* = 0.004 *t* = -0.974	0.07 (-0.07, 0.20)^d^
Mean (SD)	5.9 (4.1)	5.6 (4)	*P* = 0.331^c^	
Dryness, EULAR-SS score				
*n*	626	358	*F* = 1.997 *t* = 1.049	0.07 (-0.06, 0.20)^d^
Mean (SD)	5.8 (2.5)	6 (2.5)	*P* = 0.295^c^	
Fatigue, PROF-SF score				
*n*	618	363	*F* = 0.320 *t* = 2.548	0.17 (0.04, 0.30)^d^
Mean (SD)	3.7 (1.7)	4 (1.8)	*P* = 0.011^c^	
Pain, ESSPRI pain scale				
*n*	625	361	*F* = 2.121 *t* = 3.104	0.21 (0.08, 0.34)^d^
Mean (SD)	5.3 (2.2)	5.7 (2.1)	*P* = 0.002^c^	
Orthostatic intolerance, OGS score				
*n*	591	338	*F* = 10.521 *t* = 2.391	0.16 (0.03, 0.30)^d^
Mean (SD)	3.5 (3.2)	4.1 (3.9)	*P* = 0.017^c^	
QoL, EQ-5D-VAS score				
*n*	614	361	*F* = 0.105 *t* = -0.371	0.03 (-0.11, 0.16)^d^
Mean (SD)	60.6 (21.2)	60.1 (21.4)	*P* = 0.711^c^	
Sleepiness, ESS score				
*n*	602	342	*F* = 1.167 *t* = 1.912	0.13 (-0.26, 0.01)^d^
Mean (SD)	8.3 (4.9)	8.9 (5.1)	*P* = 0.056^c^	

BMI, body mass index; COMPASS, composite autonomic symptom scale; EQ-5D-VAS, EuroQol-5 dimension health-related quality of life scale, visual analogue scale; ESS, epworth sleepiness scale; ESSPRI, European alliance of associations for rheumatology (EULAR) Sjögren’s syndrome patient reported index; EULAR-SS, European alliance of associations for rheumatology sicca score; HADS-A, hospital anxiety and depression scale, anxiety sub-scale; HADS-D, hospital anxiety and depression scale, depression sub-scale; kg, kilograms; m, meters; OGS, orthostatic grading scale; PROF-SF, profile of fatigue and discomfort, somatic fatigue scale; SD, standard deviation; QoL, quality of life; CI, confidence interval; a: *chi-square*; b: Cramer’s V; effect sizes: small (0.1), medium (0.3) and large (0.5); c: *t*-test; d: Cohen’s d; effect sizes: small (0.2), medium (0.5) and large (0.8); -: not applicable.

**Figure 2:**
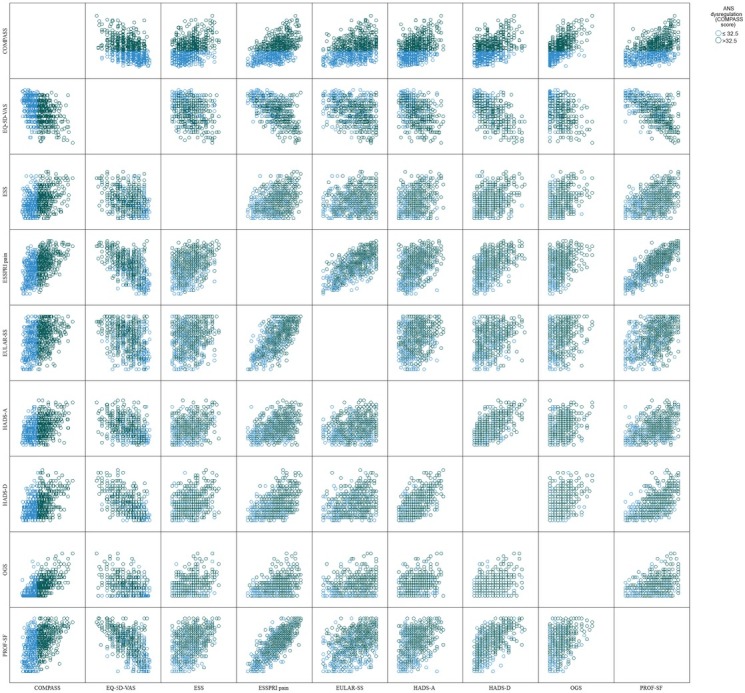
Scatter-plot matrix with PROMs scores for COMPASS completers with score ≤ 32.5 (blue circles) and with score > 32.5 (green circles).

Amongst COMPASS completers, 52% had a score > 32.5, indicative of ANS dysregulation.^[1]^ Table 3 provides a summary of socio-demographic characteristics and descriptive statistics for PROMs scores, for COMPASS completers with score ≤ 32.5 and with score > 32.5. Those with COMPASS score > 32.5, when compared to those with score ≤ 32.5, had significantly more severe scores on all PROMs (all *P* < 0.01 on *t*-test). Differences in mean scores were 3 units on the HADS-A, 2.9 units on the HADS-D, 1.4 units on the EULAR-SS, 1.3 units on the PROF-SF, 1.8 units on the ESSPRI pain scale, 3.2 units on the OGS, 14.1 units on the EQ-5D-VAS and 3.1 units on the ESS. Effect sizes were medium to large for the differences in all PROMs (Cohen’s d 0.60 to 1.19, confidence intervals on Table 3). Figure 2 provides a scatter-plot matrix with scores for all the PROMs, for COMPASS completers with score ≤ 32.5 and with score > 32.5.

**Table 3:** Clinical characteristics for COMPASS completers with score ≤ 32.5 and with score > 32.5

	COMPASS score ≤ 32.5	COMPASS score > 32.5	Effect of group	
			Comparator test	Effect size (95% CI)
Gender, *n* (%)				
*n*	303	324		
Female	283 (93)	304 (94)	*P* = 0.827^a^	0.01^b^
Age, years				
*n*	302	323	*F* = 0.024 *t* = 2.933	
Mean (SD)	59.6 (12.5)	56.7 (12.4)	*P* = 0.003^c^	0.24 (0.07, 0.39)^d^
Ethnicity, *n* (%)				
*n*	303	324		
Caucasian	287 (94.7)	308 (95.1)		
Indian	8 (2.6)	4 (1.2)		
Black	2 (0.7)	3 (0.9)		
Other	6 (2)	9 (2.8)	*P* = 0.598^a^	0.08^b^
BMI, kg/m^2^				
*n*	302	319		
			*F* = 0.817	
Mean (SD)	26.4 (5.7)	26.9 (5.6)	*t* = -1.073	0.09 (-0.07, 0.24)^d^
			*P* = 0.284^c^	
ANS function, COMPASS total	weighted score			
*n*	303	324	*F* = 43	
			*t* = -32.945	
Mean (SD)	19.8 (7.9)	48.7 (13.2)	*P* < 0.001^c^	2.60 (2.40, 2.80)
Anxiety, HADS-A score				
*n*	297	314	*F* = 10.893	
			*t* = -8.898	
Mean (SD)	6.3 (3.8)	9.3 (4.6)	*P* < 0.001^c^	0.72 (0.56, 0.88)^d^
Depression, HADS-D score				
*n*	296	318	*F* = 20.361	
Mean (SD)	4.4 (3.3)	7.3 (4.2)	*t P* = < -9.402 0.001^c^	0.76 (0.60, 0.92)^d^
Dryness, EULAR-SS score				
*n*	302	323	*F* = 5.813	
			*t* = -7.479	
Mean (SD)	5.1 (2.6)	6.5 (2.3)	*P* < 0.001^c^	0.60 (0.44, 0.76)^d^
Fatigue, PROF-SF score				
*n*	296	321	*F* = 15.790	
			*t* = -10.230	
Mean (SD)	3 (1.7)	4.3 (1.5)	*P* < 0.001^c^	0.82 (0.66, 0.99)^d^
Pain, ESSPRI pain scale				
*n*	302	322	*F* = 2.660	
			*t* = -11.115	
Mean (SD)	4.4 (2.1)	6.2 (2)	*P* < 0.01^c^	0.89 (0.73, 1.06)^d^
Orthostatic intolerance, OGS	score			
*n*	283	307	*F* = 10.800	
			*t* = -14.456	
Mean (SD)	1.8 (2.3)	5 (3)	*P* < 0.001^c^	1.19 (1.02, 1.37)^d^
QoL, EQ-5D-VAS score				
*n*	297	316	*F* = 3.937	
			*t* = 8.692	
Mean (SD)	67.9 (19.1)	53.8 (20.8)	*P* < 0.001^c^	0.70 (0.54, 0.87)^d^
Sleepiness, ESS score				
*n*	293	308	*F* = 6.421	
			*t* = -8.196	
Mean (SD)	6.7 (4.2)	9.8 (4.9)	*P* < 0.001^c^	0.67 (0.50, 0.83)^d^

BMI, body mass index; COMPASS, composite autonomic symptom scale; EQ-5D-VAS, EuroQol-5 dimension health-related quality of life scale, visual analogue scale; ESS, epworth sleepiness scale; ESSPRI, european alliance of associations for rheumatology (EULAR) Sjögren’s syndrome patient reported index; EULAR-SS, European alliance of associations for rheumatology sicca score; HADS-A, hospital anxiety and depression scale, anxiety sub-scale; HADS-D, hospital anxiety and depression scale, depression sub-scale; kg, kilograms; m, meters; OGS, orthostatic grading scale; PROF-SF, profile of fatigue and discomfort, somatic fatigue scale; SD, standard deviation; QoL, quality of life; CI, confidence interval; a: *chi-square*; b: Cramer’s V; effect sizes: small (0.1), medium (0.3) and large (0.5); c: *t*-test; d: Cohen’s d; effect sizes: small (0.2), medium (0.5) and large (0.8).

### Regression Analysis

Stepwise multiple regression identified four independent predictors of COMPASS scores: ESSPRI pain scale (β = 0.32, *P* < 0.001), EULAR-SS (β = 0.15, *P* < 0.001), HADS-A (β = 0.17, *P* < 0.001) and HADS-D (β = 0.12, *P* = 0.011). These four independent variables accounted for 34% of the variability in COMPASS scores (Supplementary Table S1).

### SEM Model

**Figure 3:**
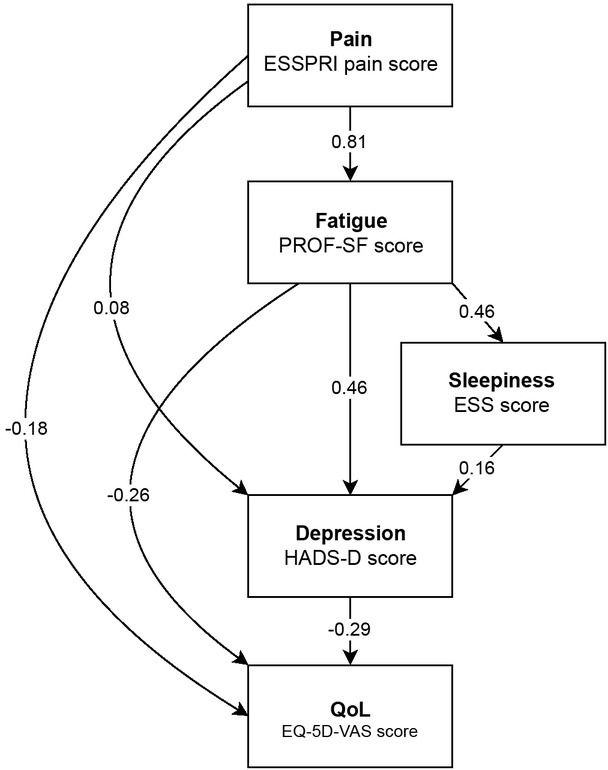
Parsimonious SEM model for the SS’s symptoms to the hypothesised outcome relationships. Coefficients alongside pathways represent what unit standard deviation change in a predictor has for the response to that driving variable. Note that the total effect of a variable on the overall quality of life outcome is the sum of the direct effects and the products of the indirect pathways to the outcome (see text).

The RMSEA for a full SEM model including the COMPASS score was very large (0.294, 95% confidence interval [CI]: 0.277-0.312) indicating a very poor fit to the data. Inspection of the COMPASS scores indicated that there were many zeroes in the data. This not only meant that the COMPASS scores were poor discriminators of outcomes, but also more prosaically that inclusion of the score offended the normality assumptions of SEM. Even when zeroes were removed, the distribution remained non-normally distributed. Removal of the COMPASS score variable and other non-significant predictors markedly improved the fit of the data to the model reducing the RMSEA to 0.052 (95% CI: 0.018-0.091) and a CFI of 0.998 indicating a good fit of the data to the model. The OGS score was significantly correlated to the COMPASS score (Pearson correlation coefficient = 0.66, *R^2^* = 0.44, *P* < 0.01). We attempted to use the OGS score as a proxy measure of ANS function but found similar issues in the data distribution. Essentially, the non-normal distribution of the COMPASS and OGS score made them inadequate for the SEM model. We also considered the use of HADS-A scores in the model alongside HADS-D but these variables were significantly correlated (Pearson correlation coefficient = 0.65, *R^2^* = 0.42, *P* < 0.001) and there was likely bi-directionality in effects. The parsimonious model is shown in Figure 3 with standardised coefficients. These coefficients represent what unit standard deviation change in the predictor would give rise to in terms of standard deviation change in the respective response variable.

In the final model, the ESSPRI pain score was a significant predictor of PROF-SF fatigue score, HADS-D depression score and overall QoL (EQ-5D-VAS score). The relationship between the ESSPRI pain score and QoL was negative, indicating that as the pain score increased QoL declined. QoL was also negatively associated with the PROF-SF fatigue score and the HADS-D depression score. The HADS-D depression score was significantly associated with the PROF-SF fatigue score, ESS sleepiness score and ESSPRI pain score. The standardised coefficient for the direct effect of the HADS-D depression score on QoL (-0.29) was of greater magnitude than the same direct effect from the PROF-SF fatigue (-0.26) and the ESSPRI pain (-0.18) scores. However, the ESSPRI pain score and PROF-SF fatigue score impacted QoL via several pathways. There was a total of 5 pathways by which the ESSPRI pain score impacted on EQ-5D-VAS QoL score, as mediated by the PROF-SF fatigue scores, the HADS-D depression score and the ESS sleepiness scores. Adding up the products of steps in a pathway over these 5 pathways [*i.e*. ((0.81 × 0.46 × 0.16 × (-0.29)) + (0.81 × 0.46 × (-0.29)) + (0.81 × (-0.26)) + (0.08 × (-0.29)) - 0.18)] led to a standardised coefficient equivalent for the overall contribution of the ESSPRI pain score to QoL of -0.54. There was a total of 3 pathways by which the PROF-SF fatigue score impacted on EQ-5D-VAS QoL score, as mediated by the ESS sleepiness and HADS-D depression scores. Adding up the products of steps in a pathway over these 3 pathways [(0.46 × 0.16 × (-0.29)) + (0.46 × (-0.29)) - 0.26] led to a standardised coefficient equivalent for the overall contribution of the PROF-SF fatigue score to QoL of -0.41.

## Discussion

This study aimed to model the role of depression in the relationships between ANS function, fatigue, and QoL in SS, whilst including other putative predictive variables. Symptoms of ANS dysregulation were common in people with SS. Participants who scored > 32.5 on the COMPASS (indicative of ANS dysregulation) had more severe self-reported depression, anxiety, dryness, fatigue, pain, sleepiness and QoL. Depression, anxiety, dryness, and pain were independent predictors of ANS function in a multiple regression. ANS function could not be included in the SEM and, when considering the interactions between all predictor variables, dryness was not a significant predictor of QoL in the SEM. Our results show that, in people with SS, depression mediates the effects of pain, fatigue and sleepiness on QoL.

All the instruments used were self-reported. We made the explicit decision not to include clinician rated instruments, such as the EULAR Sjögren’s syndrome disease activity index (ESSDAI) in our analysis. While scales such as the ESSDAI (clinician rated) and ESSPRI (patient rated) are known to not highly correlate,^[31]^ and therefore issues with multicollinearity are unlikely, they test different constructs and we chose to focus on the experiences of patients. This seems to us like a reasonable approach, as the information PROMs capture is arguably more relevant for the assessment of QoL: it has been shown to correlate well with clinician-rated scales in depression^[32]^ and to offer relevant complementary information to objective measures.^[33]^ The effect sizes for the differences between COMPASS completers and no-completers were small. Overall, the group of COMPASS completers appeared comparable to the whole cohort. An analysis of ANS symptoms was previously done on the UKPSSR,^[2]^ at a time when the available sample of COMPASS completers was about half of what we now report (317 people in total): our results are similar, in that, using multiple regression, ESSPRI and HADS-A scores are the most significant predictors of COMPASS scores. However, unlike SEM, this method does not control for the indirect effects factors may have through impact on other drivers.

Not all COMPASS completers were the same. Those who scored > 32.5 on the COMPASS (conceptualised as having ANS dysregulation) had significantly more severe scores on all other PROMs, with average differences which appear be clinically important when compared to other patient populations.^[2, 3, 4]^ In particular, the large effect sizes for the differences between groups in fatigue and orthostatic intolerance scores replicate findings in chronic fatigue syndrome, where the same COMPASS threshold was used to define ANS dysregulation.^[21]^ Conversely, it is also important to note that although symptoms of ANS dysregulation are common in people with SS, about half of COMPASS completers, with a well-established diagnosis of SS, had COMPASS scores ≤ 32.5. Therefore, it seems likely that there is inter-individual variability in ANS dysregulation amongst people of SS. This might be relevant for clinical management, as dysautonomia-directed approaches for fatigue in SS are promising. In an open pilot study of non-invasive vagus nerve stimulation (nVNS) in people with SS,^[34]^ twice daily stimulation over 26 days was significantly associated to reduction in fatigue scores and inflammatory markers. In a follow-up sham-controlled study using the same nVNS device in people with SS,^[35]^ active treatment twice daily over 54 days significantly reduced several self-reported measures of fatigue, including the PROF-SF score, which correlated significantly with electroencephalographic (EEG) measures of alpha reactivity during active stimulation, suggesting therapeutic effects related to the cholinergic system. If the effects of nVNS on fatigue in people with SS are indeed mediated by autonomic modulation, the COMPASS might be an important tool to select patients and optimize outcomes.

Given the central role of QoL as an outcome measure in our SEM, it is relevant to put the scores for EQ-5D-VAS in context: the average score in our sample is similar to samples of patients with rheumatoid arthritis in Denmark, England and Scotland.^[36]^ Our SEM model is a good representation of the non-deterministic and non-reductionist view that some factors might increase the risk of fatigue and poor QoL, but the interactions are complex and not linear.^[37]^ For example, our SEM model shows that changes to fatigue levels will have a direct impact on QoL, but also on depression, both directly and via changes to levels of sleepiness. Depression will then impact QoL directly, but also be impacted by pain, which in itself will also impact on fatigue. Therefore, a network of associations which are linked, surely more complex than the model we hypothesized and tested.

The association of ANS dysregulation with SS,^[2]^ fatigue^[21]^ and depression^[12]^ is not novel, but many fundamental questions remain unanswered. Fatigue is a core and burdensome symptom of SS and depression, whose pathophysiology is not clear. The frequency and strength of the associations between SS, depression and fatigue suggest there is a shared pathophysiology, but this remains elusive. Common research targets for fatigue include the autonomic, immune, metabolic, and neuroendocrine systems,^[37]^ as well as psychosocial and behavioural mechanisms.^[38]^ Our approach did not focus on abnormalities of a single system, but rather on the interactions of multiple common symptoms, acting as proxies for multi system involvement. Future work should include objective measures of individual systems to understand the underlying mechanisms for these links, which could provide therapeutic targets. This will require longitudinal cohort studies that combine rating scales and objective physiological measures, while recognizing the network nature of these associations. Promising targets include EEG alpha reactivity for central ANS function^[39]^ and magnetic resonance spectroscopy for muscle fatigue.^[40]^ The validity of this approach has been demonstrated in controlled trials in chronic fatigue syndrome.^[41]^

Anxiety correlates with ANS function^[2]^ and disease activity^[11]^ in SS. We considered the inclusion of anxiety alongside depression in the SEM but this poses significant methodological issues, namely the significant correlation between both measures and the bidirectionality of effects, as anxiety and depressive disorders are known to be a bidirectional risk factors for one another.^[42]^ Exploring the relationship between depression and anxiety in this population is likely to require longitudinal designs, so we can understand which develops first (depression or anxiety) and what its effects are over time in the other.

This study has several limitations. Most of the sample was female and Caucasian. We did not control for comorbid diagnosis apart from the exclusion of people with a primary diagnosis of chronic fatigue syndrome or fibromyalgia. Health related behaviours such as smoking, alcohol use and diet were not explored. ANS function measures (COMPASS) were only available in a sub-sample and could lead to bias. We also only explore the SEM as described. The relationships between variables could be different or have different directionality: for example, depression or pain could worsen ANS dysregulation and this contribute to fatigue and dryness. In the future it would be interesting to compare the outcomes from patient rated scales (as we did in this analysis), with clinician-rated scales and biomarkers, to analyse the correlations between these different measures and respective constructs, as well as the differential impact these have on clinical outcomes.

People with SS and self-reported ANS dysregulation have more severe self-rated depression, anxiety, dryness, fatigue, pain, sleepiness and QoL. Depression appears to be a mediator for the effects of pain, fatigue, and sleepiness on QoL. We were unable to further clarify the role of ANS dysregulation on these associations. Still, our results show that diagnosing and treating depression in people with SS could have direct positive impact on QoL, and significantly ameliorate the impact of fatigue and pain.

## Supplementary Material

Supplementary Materials
