# Supplementary material for: Depression in Sjögren’s syndrome mediates the relationship between pain, fatigue, sleepiness, and overall quality of life
Source: Rheumatol Immunol Res. 2023 Jul 22;4(2):78–89. doi: 10.2478/rir-2023-0012 (PMC10561071; doi:10.2478/rir-2023-0012)
Supplement: Supplementary file 1 — Supplementary Materials [file rir-2023-0012_supp.pdf]

Supplementary Materials

Supplementary Table S1: Results from stepwise multiple regression analysis using COMPASS total score as dependent variable

	**Unstandardised coefficients**		**Standardised coefficient**	*t*	**Significance**
	**B**	**SE**	**β**		
Constant	7.821	1.736		4.505	< 0.001
ESSPRI pain	2.596	0.338	0.317	7.680	< 0.001
HADS-A	0.687	0.174	0.171	3.937	< 0.001
ESS	0.551	0.140	0.148	3.938	< 0.001
HADS-D	0.544	0.213	0.121	2.556	0.011
$R^2 = 0.341$					

Excluded variable (stepwise): EULAR-SS, PROF-SF and EQ-5D-VAS.

COMPASS, composite autonomic symptom scale; EQ-5D-VAS, EuroQol-5 dimension health-related quality of life scale, visual analogue scale; ESS, epworth sleepiness scale; ESSPRI, European alliance of associations for rheumatology (EULAR) Sjögren's syndrome patient reported index; EULAR-SS, European alliance of associations for rheumatology sicca score; HADS-A, hospital anxiety and depression scale, anxiety sub-scale; HADS-D, hospital anxiety and depression scale, depression sub-scale; PROF-SF, profile of fatigue and discomfort, somatic fatigue scale; SE, standard error.
